# External validation of the ACC/AHA ASCVD risk score in a Colombian population cohort

**DOI:** 10.1038/s41598-023-32668-4

**Published:** 2023-04-15

**Authors:** Carlos Daniel Rodríguez-Ariza, Alfredo Cabrera-Villamizar, Astrid Lorena Rodríguez-Pulido, Santiago Callegari, Natalia Alejandra Ossa Rodríguez, Mónica Pinilla-Roncancio, Sergio Mauricio Moreno López, Carlos Andrés Sánchez-Vallejo

**Affiliations:** 1 Critical Care Section, Internal Medicine Department, Fundación Santa Fe de Bogotá, Bogotá, Colombia; 2 Internal Medicine Department, Fundación Santa Fe de Bogotá, Bogotá, Colombia; 3 Faculty of Medicine, Universidad de los Andes, Bogotá, Colombia; 4 Cardiovascular Department, Beth Israel Deaconess Medical Center, Boston, USA; 5 Cardiology Section, Internal Medicine Department, Fundación Santa Fe de Bogotá, Bogotá, Colombia

**Keywords:** Cardiology, Disease prevention

## Abstract

No cardiovascular risk score has included Latin American patients in its development. The ACC/AHA ASCVD risk score has not been validated in Latin America; consequently, its predictive capacity in the population of the region is unknown. The aim of this study is to evaluate the discrimination capacity and calibration of the ACC/AHA ASCVD score to predict the 10-year risk of a cardiovascular event in a primary prevention cohort followed in a Colombian hospital. A retrospective cohort study was conducted in primary prevention patients belonging to an intermediate/high-risk and low-risk cohort without established atherosclerotic disease. Cardiovascular risk was calculated at inclusion. The calibration was analyzed by comparing observed and expected events in the different risk categories. A discrimination analysis was made using the area under the ROC curve and C statistic. A total of 918 patients were included—202 from the intermediate/high-risk and 716 from the low-risk cohort. The median cardiovascular risk was 3.6% (IQR 1.7–8.5%). At the 10-year follow-up, 40 events (4,4%) occurred. The area under the ROC curve was 0.782 (95% CI 0.71–0.85). The Hosmer–Lemeshow test did not show differences between expected and observed events. The ACC/AHA ASCVD score is calibrated and has good discrimination capacity in predicting 10-year risk of cardiovascular events in a Colombian population.

## Introduction

Cardiovascular disease (CVD) is considered the main cause of morbidity and mortality in the world, with more than 18.6 million attributable deaths in 2019^1–3^, which is equivalent to almost 400 million disability-adjusted life years (DALYs)^4^. Of these deaths, 80% occur in low- and middle-income countries, including Latin American countries, where prevalence continues rising^5,6^. Additionally, the cost to the health system is highly relevant. In developing countries, such as Colombia, it is estimated that coronary heart disease represents more than 4,000 United States dollars (USD) per patient per year to the health system, a figure that increases to 6,000 USD in the case of stroke^7^.

The late and inadequate implementation of measures to prevent cardiovascular events is one of the main causes of this disease's increased prevalence, mortality, and high costs^8,9^. One of the most important preventive strategies is the estimation of cardiovascular risk, which allows to predict the probability of an individual experiencing a major cardiovascular event in the next ten years. Calculated risk is then used to make prompt therapeutic decisions regarding pharmacological and non-pharmacological interventions^10^. Some tools recommended by scientific societies to assess cardiovascular risk include the Framingham score^11^, PROCAM (Prospective Cardiovascular Münster)^12^, the European SCORE (System for Cardiac Operative Risk Evaluation)^13^, and the ACC/AHA ASCVD (American Heart Association/Atherosclerotic cardiovascular disease) score^14^. However, none of these scores included Latin American population in the development of their models. Neither have external validation studies in this population^15^. Due to this lack of inclusion, using any of these models in Latin American population could lead to an over- or underestimation of cardiovascular risk^16^. Therefore, wrong treatment decisions are made, and timely implementation of preventive strategies are delayed.

A study conducted in Colombia found that the Framingham and PROCAM scores overestimate the cardiovascular risk, particularly in the highest risk categories^17^. This could be explained by the time in which these scores were developed and the inclusion of patients with low control of risk factors and a high burden of poorly controlled comorbidities^18^. This instance highlights the importance of an external validation process of a score in the current population^19^.

The ACC/AHA ASCVD risk score is easy to use, was developed using more recent data on risk factors and comorbidities and reflects better the status of cardiovascular risk factors and their contribution to the disease development. This score, unlike its predecessors, allows the simultaneous calculation of the risk of acute fatal and non-fatal myocardial infarction, and stroke^20^. In addition, it is recommended in the AHA guidelines to define different prevention strategies, such as initiation of aspirin, statins use, and blood pressure and LDL (low-density lipoprotein) cholesterol goals^21^.

## Methods

### Aim of study

This study intends to evaluate the behavior of ACC/AHA ASCVD risk score in terms of discrimination and calibration for predicting cardiovascular risk in a sample of primary prevention cohort followed in a Colombian hospital.

### Study design

This retrospective cohort study was conducted from July 2021 to May 2022 in patients without established cardiovascular disease evaluated at the Hospital Universitario Fundación Santa Fe de Bogotá (FSFB) between 2009 and 2010. FSFB is a tertiary care center located in Bogotá, the capital of Colombia which provides care to patients from different regions across the country, mainly from private health insurances.

The risk of atherosclerotic cardiovascular events was calculated with baseline data, and, subsequently, the occurrence of major cardiovascular events within ten years was observed. The study was approved by the ethics committee at Fundación Santa Fe de Bogotá. Our study was performed in accordance with the Declaration of Helsinki and established regulations. Informed consent was obtained from all participants before including them in the study.

### Data source

Data from two cohorts of patients seen in outpatient special care programs were used. The first cohort (intermediate/high-risk) corresponds to a special care program composed of 421 patients belonging to the hypertension and diabetes program of the hospital in 2009 and 2010. The second cohort (low risk) is composed of 2334 patients seen in the FSFB executive screening program in 2009 and 2010.

According to the study's inclusion criteria, the patients were required to be part of the hypertension and diabetes program or the executive screening program (a preventive medical screening service available for the general population) in 2009 or 2010. The clinical history of each of the patients in these two programs was reviewed to determine the first consultation at FSFB, where sufficient data was collected to estimate their cardiovascular risk using the ACC/AHA ASCVD score. The cardiovascular risk estimation was performed only in patients who met the eligibility criteria. Subsequently, the medical records of the included patients were reviewed to verify whether they experienced any major cardiovascular events in the ten years following the date of risk calculation.

Patients who did not have 10-year follow-up data, were contacted by telephone or email to inquire about the occurrence of cardiovascular events in the specified 10-year period. All information collected from both cohorts was stored in REDCap. Access to said file, as well as to the medical records, was exclusive to the study investigators.

### Participants

Patients were selected from the two cohorts based on the following inclusion criteria: Colombian patients aged 40–79 years, with no history of previous major cardiovascular events, LDL-c levels less than 190 mg/dl, and who had sufficient information to calculate their 10-year cardiovascular risk. The exclusion criteria were: patients whose cardiovascular events in the 10 years after risk calculation were unable to be verified, evidence of other forms of previous atherosclerotic cardiovascular disease (transient ischemic attack, unstable angina, angioplasty, coronary artery bypass grafting (CABG), or peripheral arterial disease) at the time of risk calculation, or statin use at the time of risk calculation. Patients taking statins were excluded to avoid bias in calculating cardiovascular risk by lowering non-HDL cholesterol levels, therefore, generating a modification of the estimated baseline risk. A non-probabilistic, consecutive sampling was carried out, which included all the subjects belonging to the databases that met the inclusion criteria. Only patients with full information for risk calculation and 10 years follow up were included for analysis.

### Outcomes

The primary endpoint was the occurrence of a major cardiovascular event. The major cardiovascular events included were fatal and non-fatal acute myocardial infarction (AMI) based on the fourth universal definition of AMI^22^, and fatal and non-fatal stroke (signs and symptoms of neurological deficit, compatible findings on computed tomography and/or cerebral nuclear magnetic resonance or need for endovascular interventions). The information about outcomes was collected from medical records and analyzed separately by each one of the investigators who assessed the validity of the diagnosis according to the previous definitions. Any discordance during the process was discussed by all the authors and only patients who were eligible for the study by consensus of all authors were included.

### Independent variables

The independent variables were: age (on the date of risk calculation), sex, race (Afro-Colombian vs white/other), total cholesterol, HDL cholesterol, systolic blood pressure, treatment for high blood pressure (Yes/No), smoking (Yes/No), and diabetes mellitus (Yes/No).

### Sample size

According to the recommendation made by authors such as Carretero, between 5 and 10 people are required for each item that makes up the score^23^. In this case, the ACC/AHA ASCVD score is made up of nine items. Therefore, a total of 90 participants is required. This number was widely exceeded for both cohorts. Patients who did not have complete information were excluded, so there are no missing variables of interest.

### Statistical analysis

Initially, a descriptive analysis of the included population was performed using the Stata17® program^24^. Continuous variables are reported as means and standard deviation, while categorical variables are reported as frequencies and proportions. The characteristics of the total population, as well as of each cohort separately, were described. The official risk calculator available on the *American Heart Association* website was used to calculate cardiovascular risk https://tools.acc.org/ldl/ascvd_risk_estimator/index.html#!/calulate/estimator/.

Calibration (coincidence between the expected and observed probability of cardiovascular events in the population) was evaluated using a multivariate logistic regression model in which the predictor variables were those included in the original model (age, sex, race, total cholesterol values, HDL cholesterol levels, systolic blood pressure, the use of an antihypertensive medication, smoking status, and diagnosis of diabetes mellitus). The interaction between these variables was evaluated at the time of model analysis. A penalized regression was used so as not to exclude data that had fewer observations in the model (e.g. Afro-Colombian race). Subsequently, the predicted events were compared with the observed events in four different risk categories (< 5%, 5–7.5%, 7.5–20%, and > 20%) according to the AHA guidelines cut-off points^21^. A Hosmer Lemeshow test (HL) was used to evaluate the goodness of fit of the model. This chi-square based test is used for binary response variables (cardiovascular event or not) and evaluates how well the data fits the model. A small HL number, commonly joined with a p value greater than 0.05, means that there is not a statistically significant difference between expected and observed events. In that case, the overall model fit is good. By choosing only 4 subgroups, the power of the HL test is greater than it could be if the subgroups were deciles of risk. In addition, in samples greater than 400, this test has good power for finding poor calibration.

Discrimination (the ability of the score to differentiate individuals who will have a cardiovascular event from those who will not) was evaluated using the C statistic, or the area under the ROC curve. A C statistic higher than 0.75 is considered good. The sensitivity, specificity, and predictive values results were analyzed for a cut-off point of 20%, which corresponds to the limit for classifying high-risk patients.

The results obtained were analyzed for the total population of the two cohorts. The behavior of the score was also analyzed in each cohort separately (intermediate/high risk vs. low risk). Finally, a sensitivity analysis was carried out with the race variable to evaluate possible changes in the diagnostic performance of the score.

## Results

### Patients characteristics

**Figure 1:**
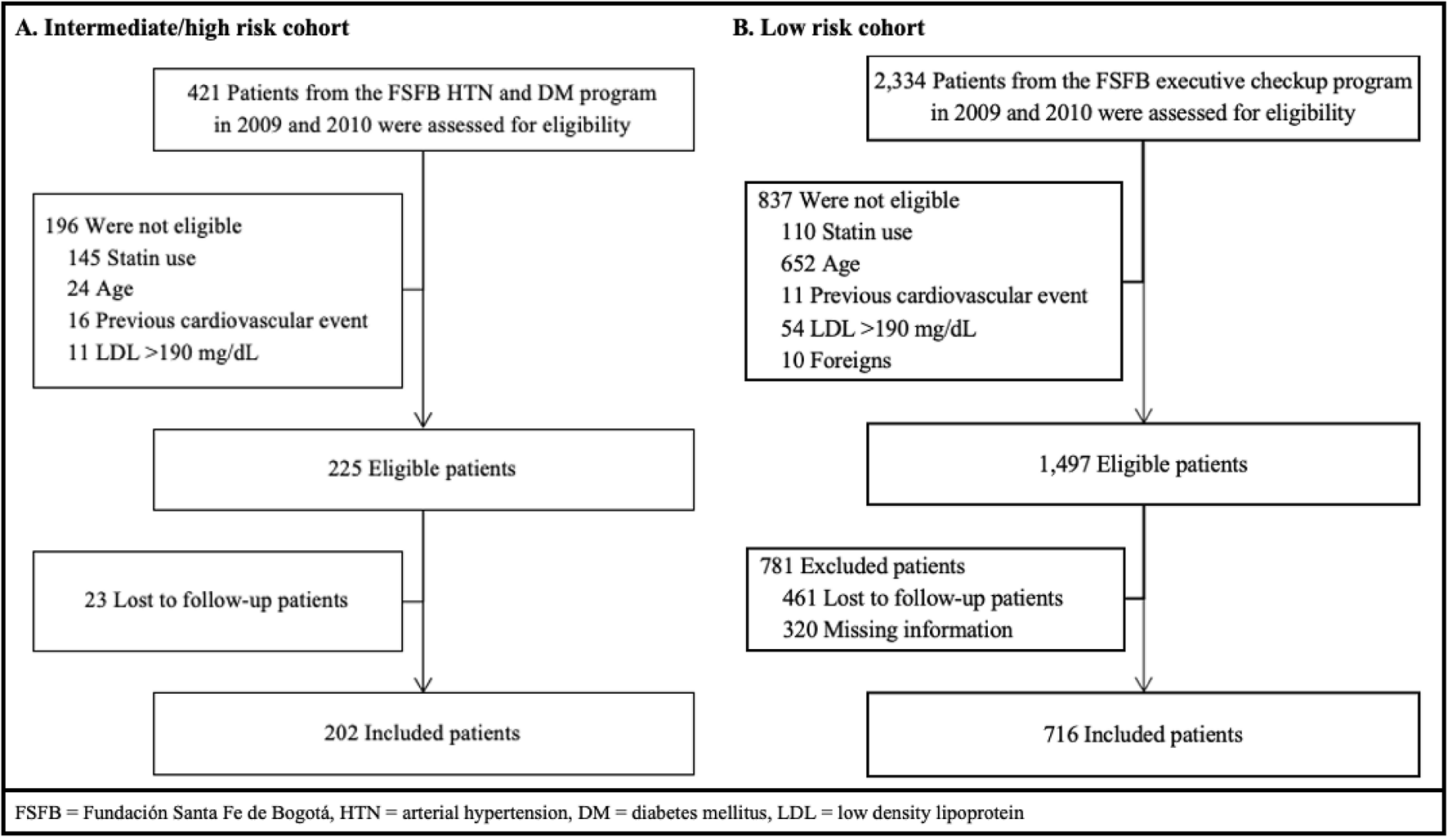
Flowchart of the patients included in the study.

The medical records of 2,755 eligible patients (421 from the hypertension and diabetes program and 2,334 from the executive screening program) were reviewed. After reviewing the inclusion and exclusion criteria as well as collecting the necessary information to verify the 10-year follow-up, a total of 918 patients were included in the study (Fig. 1). The main reasons for excluding patients were age (676 patients) and use of statins at the time of assessment (255 patients). On the other hand, 465 patients met eligibility criteria but did not have a 10-year follow-up at the institution, and it was not possible to contact them to verify the development of events. Table S1 of the supplementary material shows the characteristics of patients lost to follow-up at 10 years. They were individuals with a lower mean age (50.5 years, SD 8.2), a lower prevalence of diabetes (4.3%), and lower rates of high blood pressure on treatment (14.4%). The cardiovascular risk calculated in the patients without follow-up at 10 years showed no significant difference compared to the risk of the included patients (*p *= 0.99).

Table 1 shows the baseline characteristics by sex of the total population included in the study and for each cohort. The 10-year cardiovascular risk calculated by the ACC/AHA ASCVD score does not have a normal distribution, so it is reported with median and interquartile range. The population was composed of 918 patients, of which 64.2% were male. Less than 1% of individuals self-identified as black. Regarding the prevalence of cardiovascular risk factors in the total cohort, 25.6% of the patients received antihypertensive treatment, with a higher proportion of women being treated than men (38% vs 18.7% *p *< 0.001). The prevalence of diabetes mellitus was similar in both sexes. Total cholesterol values were similar in both sexes, while women had higher HDL levels (52.1 vs 42.2 *p *< 0.001). Regarding comorbidities not included in the risk model, more than half of the patients were overweight or obese. The prevalence of chronic kidney disease (1.5%) and inflammatory diseases (2.8%) was very low in the included population.

**Table 1:** Baseline characteristics.

*Characteristic*	Total cohort				Intermediate/high risk cohort			Low-risk cohort		
	Total (n = 918)	Men (n = 589)	Women (n = 329)	*p* value	Total (n = 202)	Men (n = 85)	Women (n = 117)	Total (n = 716)	Men (n = 504)	Women (n = 212)
Age (years), median (SD)	51.7 (9.2)	51.3 (8.8)	52.4 (9.7)	0.09**	59.1 (9.6)	60.1 (10.1)	58.3 (9.2)	49.6 (7.9)	49.8 (7.7)	49.1 (8.3)
Afrocolombian race, n (%)	9 (1)	3 (0.5)	6 (1.8)	0.053*	2 (1)	1 (1.2)	1 (0.8)	7 (1)	2 (0.4)	5 (2.4)
Smoking. n (%)	91 (9.9)	54 (9.2)	37 (11.2)	0.31*	13 (6.4)	3 (3.5)	10 (8.5)	78 (10.9)	51 (10.1)	27 (12.7)
Diabetes mellitus, n (%)	113 (12.3)	69 (11.7)	44 (13.4)	0.46*	97 (48)	59 (69.4)	38 (32.5)	16 (2.2)	10 (2)	6 (2.8)
Antihypertensive treatment, n (%)	235 (25.6)	110 (18.7)	125 (38)	< 0.001*	156 (77.2)	59 (69.4)	97 (82.9)	79 (11)	51 (10.1)	28 (13.2)
TC (mg/dl), median (SD)	206.7 (34)	207.1 (34.7)	206 (32.8)	0.62**	199.2 (34.7)	189.9 (37.8)	205.9 (30.7)	209 (33.6)	210 (33.4)	206 (33.9)
HDLc (mg/dl), median (SD)	45.8 (12.1)	42.2 (9.1)	52.1 (14)	< 0.001**	45.9 (14.6)	40.6 (9.2)	49.8 (16.5)	45.7 (11.3)	42.5 (9.1)	53.3 (12.3)
SBP (mmHg), median (SD)	122.4 (17.1)	123.6 (15.9)	120.2 (18.9)	0.005**	124.9 (16.5)	125.5 (15.8)	124.4 (17.1)	121.7 (17.2)	123.2 (15.9)	117.9 (19.5)
Overweight, n (%)	409 (44.5)	294 (49.9)	115 (34.9)	< 0.001*	107 (53)	53 (62.3)	54 (46.1)	302 (42.2)	241 (47.8)	61 (28.8)
Obesity, n (%)	150 (16.3)	90 (15.3)	60 (18.2)	< 0.001*	53 (26.2)	18 (21.2)	35 (29.9)	97 (13.5)	72 (14.3)	25 (11.8)
CKD, n (%)	14 (1.5)	11 (1.9)	3 (0.9)	0.26*	11 (5.5)	9 (10.6)	2 (1.7)	3 (0.4)	2 (0.4)	1 (0.5)
CID, n (%)	26 (2.8)	11 (1.9)	15 (4.6)	0.02*	18 (8.9)	6 (7.1)	12 (10.3)	8 (1.1)	5 (1)	3 (1.4)
AHA/ASCVD score, median (IQR)	3.6 (1.7–8.5)	4.55 (2.5–9.6)	2.1 (0.9–6.05)	< 0.001***	8.25 (3.4–18.5)	14.7 (7.3–29.2)	5 (2.7–11.2)	3.1 (1.5–6.5)	3.9 (2.3–7.8)	1.1 (0.5–2.5)
Observed CV events, n (%)	40 (4.4)	27 (4.6)	13 (3.9)	0.65*	21 (10.4)	9 (10.6)	12 (10.3)	19 (2.7)	18 (3.6)	1 (0.5)

*TC* Total cholesterol, *HDLc* High density lipoprotein cholesterol, *SBP* Systolic blood pressure, *CKD* Chronic kidney disease, *CID* Chronic inflammatory diseases, *CV* Cardiovascular.

*p-value by X2. ** *p*-value by t-Student. *** *p*-value by Mann Whitney.

**Figure 2:**
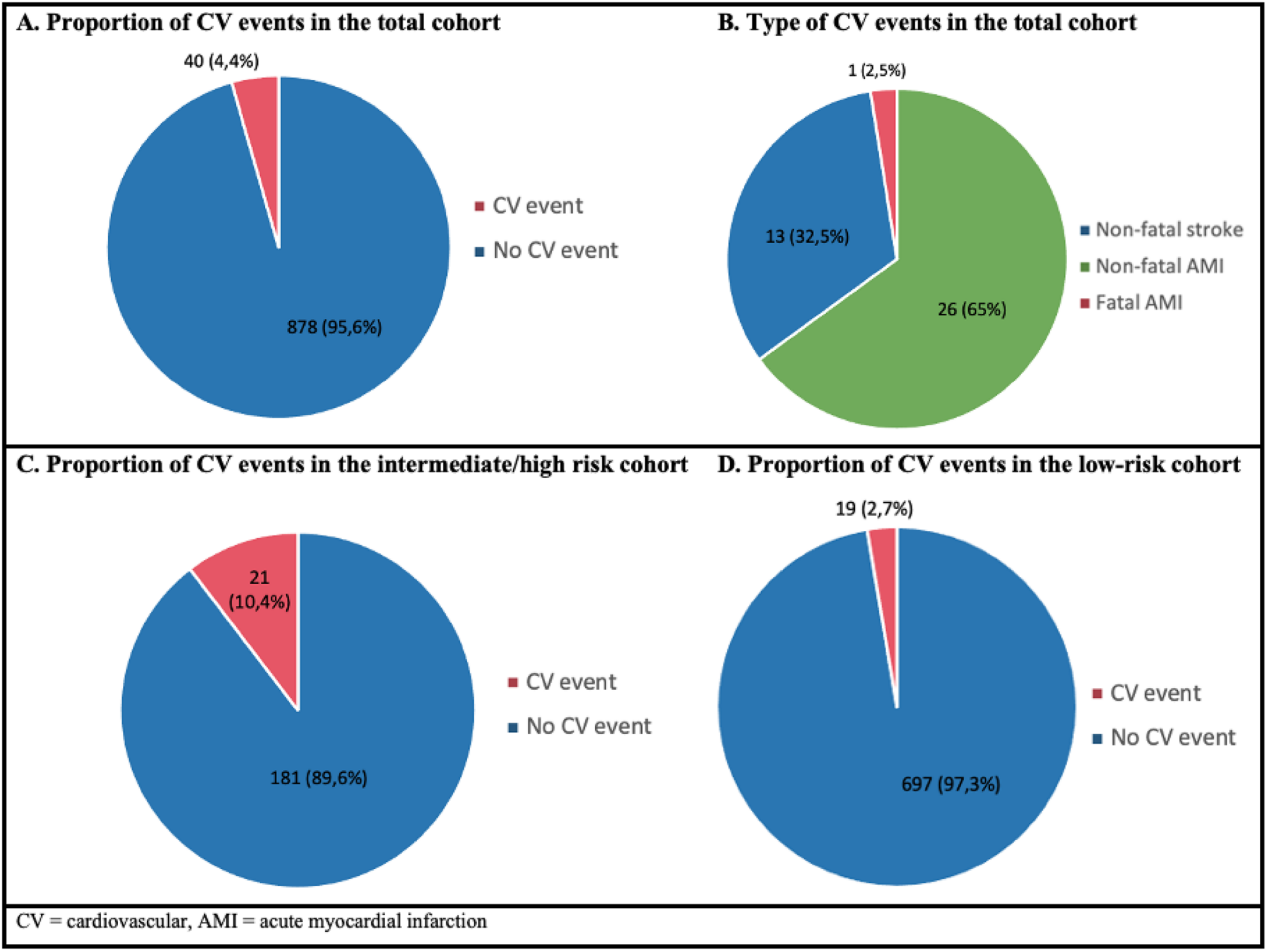
Proportion and type of events for the total cohort (**A**,**B**). Proportion of cardiovascular events in the high (**C**) and low-risk (**D**) cohorts.

The median cardiovascular risk calculated for these patients was 3.6% in the total cohort (IQR 1.7–8.5), and it was significantly higher in men than women in each of the cohorts (*p *< 0.001 by Mann Whitney). In the 10-year follow-up, only 40 patients (4.4%) had a cardiovascular event, which coincides with the 3.6% risk estimated by the ACC/AHA ASCVD score for the entire cohort (Fig. 2A). Of the 40 events, 13 were non-fatal stroke, 26 were non-fatal AMI and 1 event was fatal AMI (Fig. 2B). When discriminating the proportion of events in each cohort, it was found that the intermediate-high risk cohort had an event proportion of 10.4%, while in the low-risk cohort, the proportion was 2.7%. In both cases, the proportion of events is consistent with the median risk calculated by the scale (8.25% and 3.1% for the intermediate/high-risk cohort and the low-risk cohort, respectively).

### Score validation

Table 2 shows the distribution of patients in each of the risk categories as well as the number and proportion of individuals who presented a cardiovascular event in each category. Nearly 80% of all cardiovascular events occurred in the intermediate and high-risk categories. In the low and borderline risk categories, the proportion of events did not exceed 2%. In the intermediate category, 12.8% of patients presented a cardiovascular event within the 10-year follow-up period, while in high-risk patients, the proportion was 9.6%.

**Table 2:** Observed and expected cardiovascular events according to the risk category in the total cohort.

AHA/ASCVD risk category	Number of patients	Observed CV events (%)	Expected CV events (%)	HL
Low risk (< 5%)	540	7 (1.3)	7.5 (1.4)	0.03
Borderline risk (5–7.5%)	116	2 (1.7)	2.5 (2.1)	0.09
Intermediate risk (7.5–20%)	179	23 (12.8)	23.4 (13.1)	0.01
High risk (> 20%)	83	8 (9.6)	8.4 (10.1)	0.02
Total	918	40 (4.4)	41.7 (4.5)	0.15*

*CV* Cardiovascular, *HL* Hosmer Lemeshow statistic. *p-*value* = 0.99*.*

**Figure 3:**
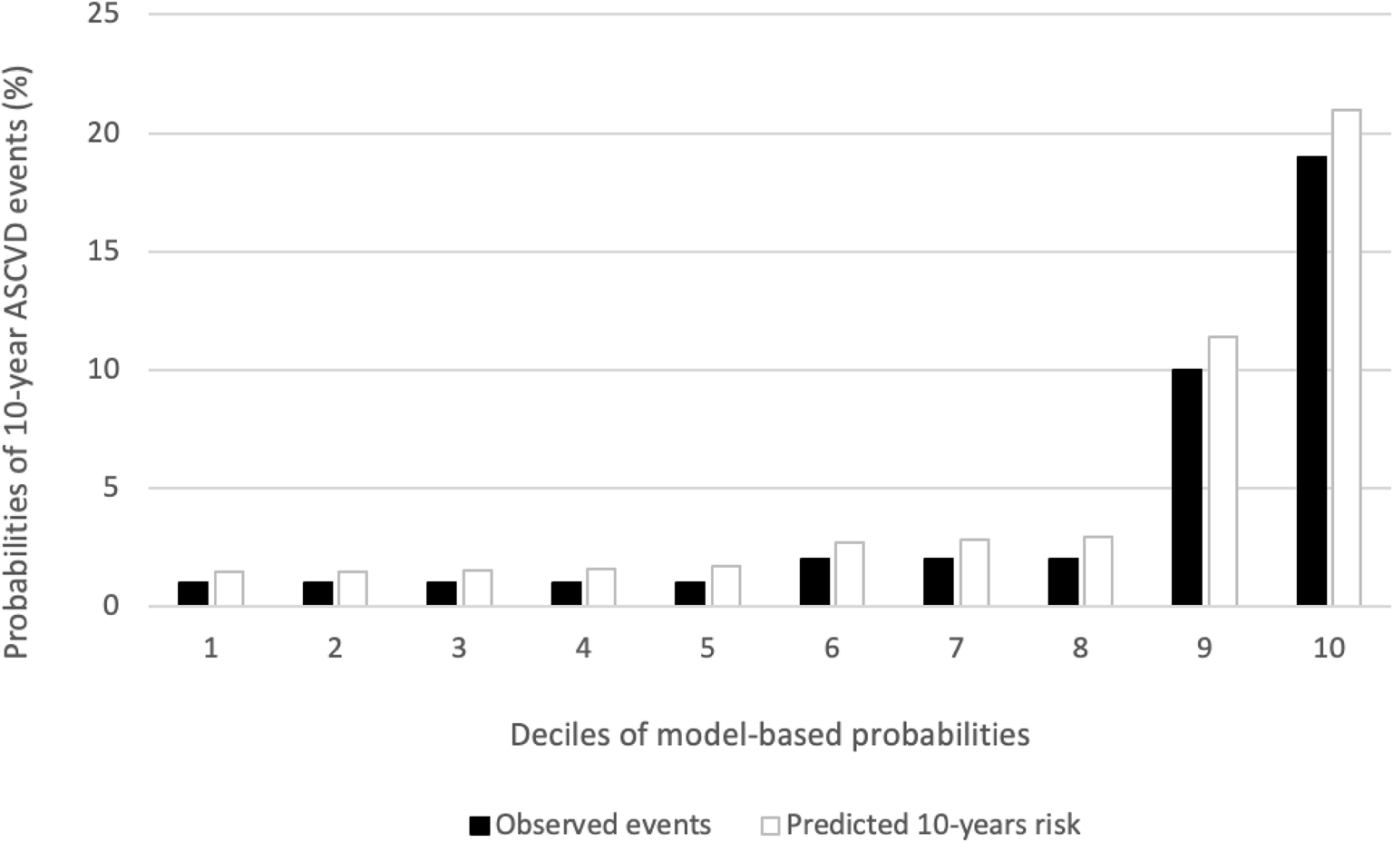
Observed and expected cardiovascular events in each decile category.

**Figure 4:**
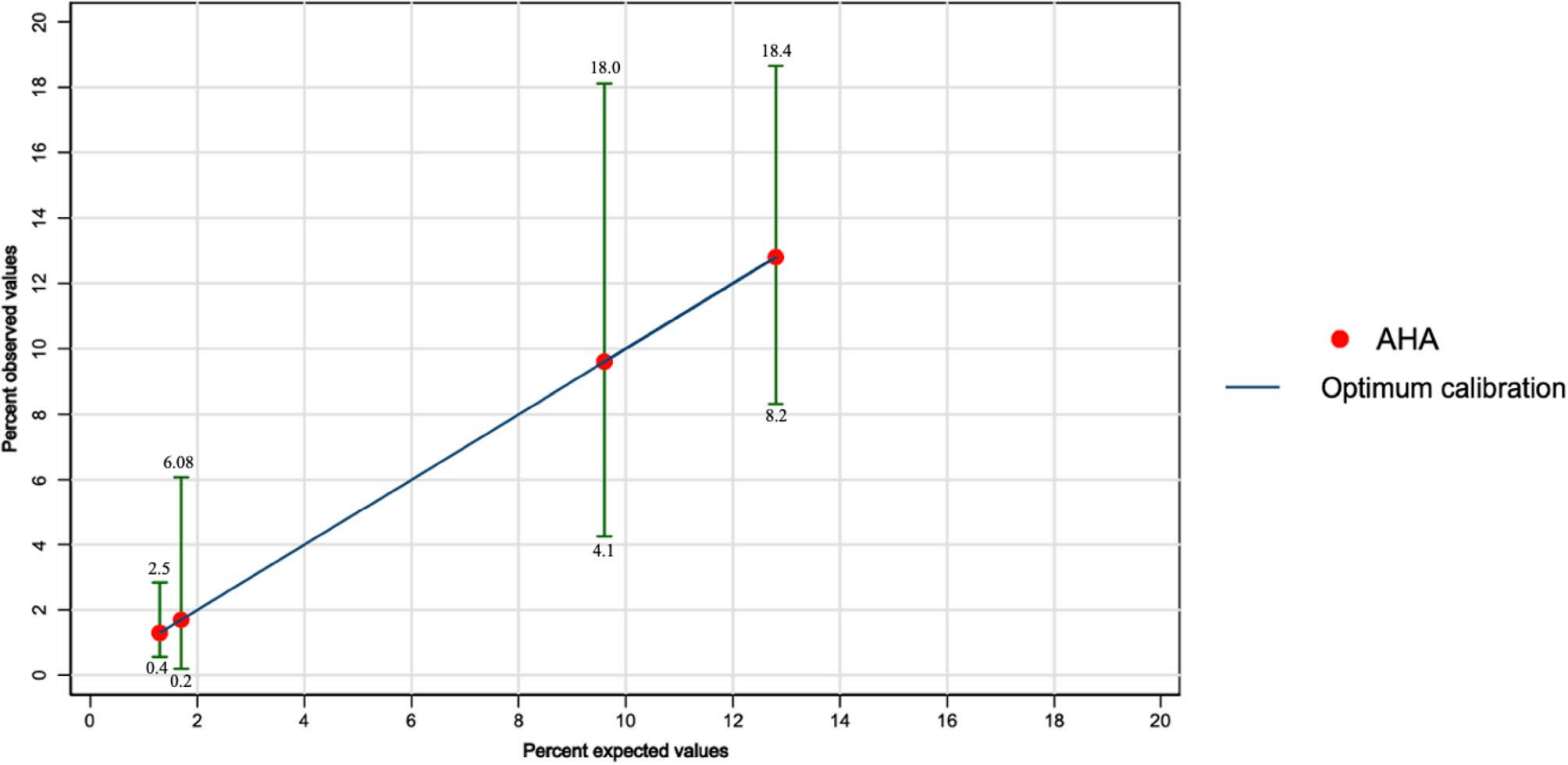
Observed and expected cardiovascular events in the total cohort. The straight line shows a test with optimum calibration (the higher the expected event rate, the higher the observed event rate). In this case, a very good calibration was found as there is no difference between expected and observed event rates.

The Hosmer Lemeshow goodness-of-fit statistic was calculated. A statistic of 0.15 was obtained with a *p-*value = 0.99, which indicates that no statistically significant difference was found between the observed and expected events. Neither was found between the observed and expected events in each of the risk categories separately (Table 2). A second analysis was performed according to deciles of risk, which revealed no difference between expected and observed events in each category (HL 1.1 *p *= 0.99) (Fig. 3). Most of the events occurred in the higher deciles of risk. In general, the score predicted a greater number of events than occurred. The ratio of expected/observed events is 1.05 for the total patient cohort (Fig. 4).

**Figure 5:**
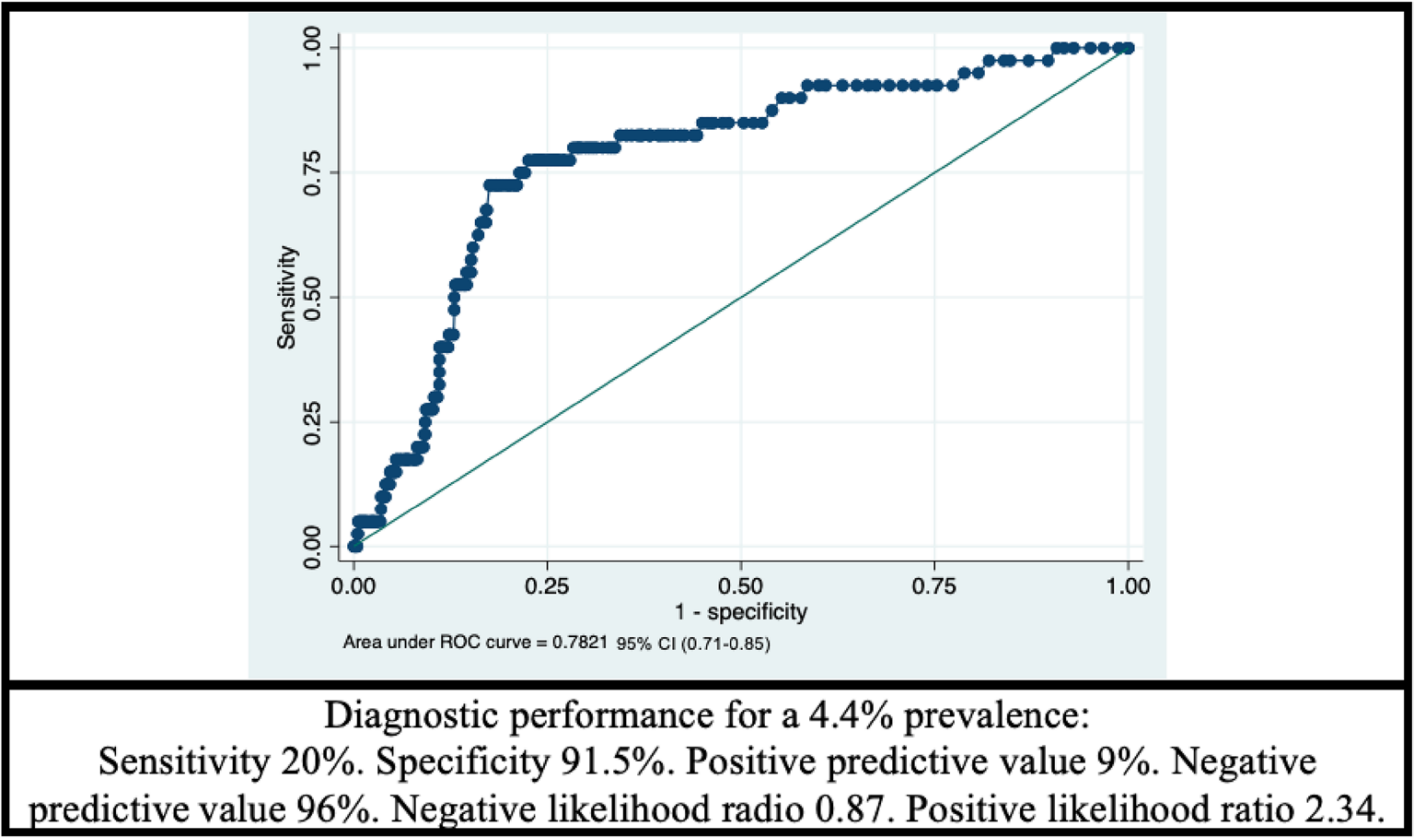
Area under the ROC curve of the AHA/ASCVD cardiovascular risk scale in the total cohort.

To evaluate the discrimination of the ACC/AHA ASCVD score, the area under the ROC curve of the model was calculated, and a value of 0.782 was obtained (Fig. 5). Additionally, the diagnostic performance of the score was estimated by assigning a cut-off point of 20%, which classifies high-risk patients. A sensitivity of only 20% and a specificity of 91.5% were obtained. This means a positive predictive value of 9% and a negative predictive value of 96% (estimated with a prevalence of 4.4% that was found in the study).

### Behavior of the ACC/AHA ASCVD score in an intermediate/high-risk cohort vs. a low-risk cohort

The cohort of patients from the hypertension and diabetes program had a high prevalence of cardiovascular risk factors (diabetes mellitus: 48% and hypertension under treatment: 77%) (Table 1). This coincides with a median cardiovascular risk of 8.25% and an event rate of 10.4% (Fig. 2C), which are above the values found in the total cohort. The low-risk cohort corresponded to the executive screening patients and had a very low prevalence of cardiovascular risk factors. Only 2.2% of those patients had diabetes mellitus, and the percentage of patients who received treatment for high blood pressure was 11%. In this cohort, the median cardiovascular risk was 3.1% with an event rate of 2.7% (Fig. 2D), which is the same proportion of events reported from the 10-year follow-up in the total cohort (Table 1).

### Calibration and discrimination in each cohort

Tables 3 and 4 show the proportion of events in each risk category. In the intermediate/high-risk cohort, 23% of patients had an estimated risk greater than 20%; however, most events occurred among patients classified as intermediate risk. In contrast, in the low-risk cohort, the highest proportion of events occurred in patients classified as high risk (13.5%). The Hosmer Lemeshow test showed that there was no significant difference between the observed and expected events (HL 0.26 in the intermediate/high-risk cohort, *p *= 0.99 and HL 0.28 in the low-risk cohort, *p *= 0.9). The ratio of expected/observed events was 1.09 in the low-risk cohort while in the intermediate/high-risk cohort it was 1.1.

**Table 3:** Observed and expected cardiovascular events according to the risk category in the intermediate/high risk cohort.

AHA/ASCVD risk category	Number of patients	Observed CV events (%)	Expected CV events (%)	HL
Low risk (< 5%)	69	4 (5.8)	4.4 (6.4)	0.05
Borderline risk (5–7.5%)	28	1 (3.6)	1.4 (5.2)	0.15
Intermediate risk (7.5–20%)	59	13 (22)	13.3 (22.5)	0.01
High risk (> 20%)	46	3 (6.5)	3.4 (7.5)	0.06
Total	202	21 (10.4)	22.6 (11.2)	0.26*

*CV* Cardiovascula*r, HL* Hosmer Lemeshow statistic. *p-value =* 0.99.*

**Table 4:** Observed and expected cardiovascular events according to the risk category in the low-risk cohort.

AHA/ASCVD risk category	Number of patients	Observed CV events (%)	Expected CV events (%)	HL
Low risk (< 5%)	471	3 (0.6)	3.5 (0.7)	0.07
Borderline risk (5–7.5%)	88	1 (1.1)	1.5 (1.7)	0.16
Intermediate risk (7.5–20%)	120	10 (8.3)	10.4 (8.7)	0.02
High risk (> 20%)	37	5 (13.5)	5.4 (14.5)	0.03
Total	716	19 (2.7)	20.7 (2.9)	0.28*

*CV* Cardiovascular, *HL* Hosmer lemeshow statistic. *p-value = 0.99.

**Figure 6:**
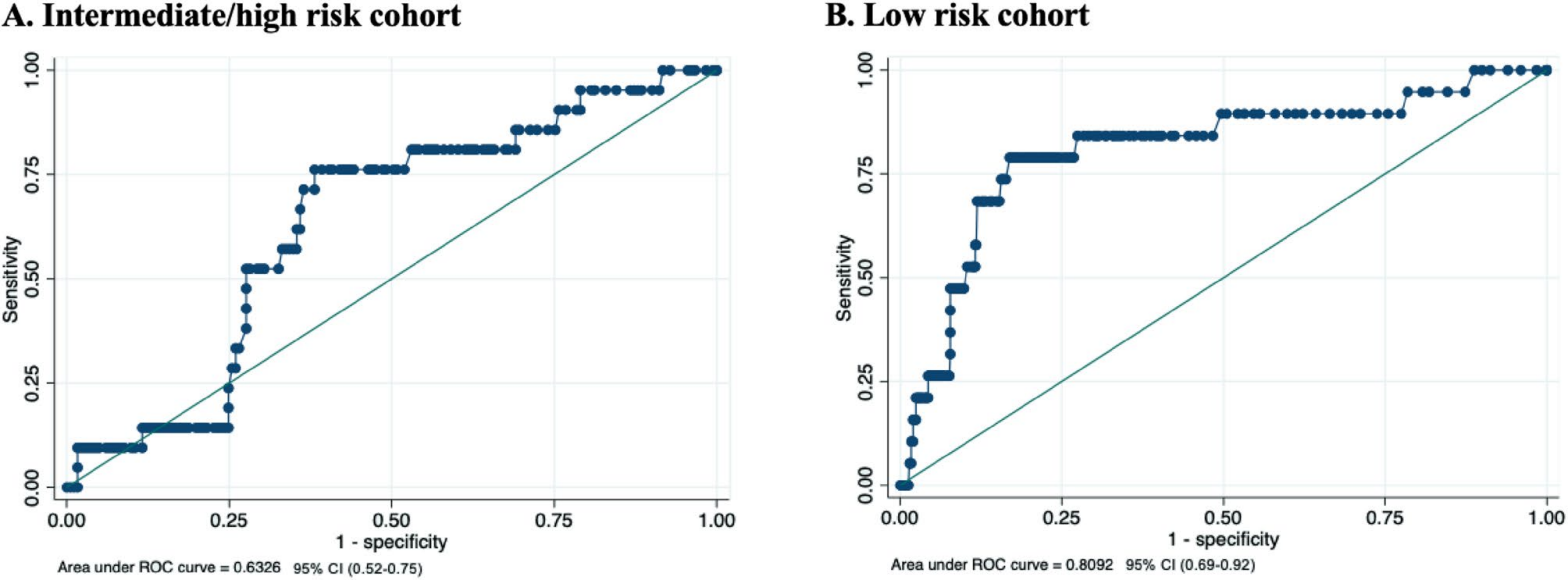
Area under the ROC curve of the AHA/ASCVD cardiovascular risk scale.

The area under the ROC curve was calculated separately for each cohort's cardiovascular risk scale. In the low-risk cohort, the area under the ROC curve is higher (0.809) than in the intermediate/high-risk cohort (AUC ROC 0.632) (Fig. 6A-B).

### Sensitivity analysis

A sensitivity analysis was performed excluding the 9 Afro-Colombian patients, finding that the median cardiovascular risk of the population remained constant. No changes were observed in the behavior of the score in terms of calibration (the C statistic did not change) or discrimination (HL p value = 0.99). There were also no significant changes in sensitivity or specificity. Subsequently, this procedure was repeated using white/other coefficients for the entire cohort, obtaining similar results.

## Discussion

The present study evaluated the behavior of the ACC/AHA ASCVD cardiovascular risk score in a primary prevention cohort in a single center in Bogotá, Colombia, obtaining data from two sources: the hypertension and diabetes program (known as the intermediate-high risk cohort) and the executive screening program (referred to as the low-risk cohort).

The diagnostic performance of the ACC/AHA ASCVD score was evaluated through a calibration and discrimination analysis which found that the higher the predicted risk, the higher the proportion of cardiovascular events. This is reflected in the fact that 79% of reported events occurred in patients with intermediate (> 7.5% of calculation) and high risk (> 20% of calculation). The HL test showed that the score is calibrated and that there is no difference between expected and observed events, with a ratio of expected/observed events of 1.05, indicating a slight overestimation of risk with no statistical significance. Overestimation of risk has been a global finding in similar studies, in which overestimations greater than this have been found, up to 75–150%^25–28^.

It has been suggested that the discrepancy between predicted risk and observed events in current cohorts is due to the use of modifying therapies such as aspirin, statins, and revascularization. However, after adjusting for the use of statins, the score continues to overestimate the risk^15,30^; thus, the overestimation of risk could be explained by other factors.

To date, this study is the first in Latin America that evaluates the discrimination of the ACC/AHA ASCVD score, finding a good discrimination capacity. The discrimination capacity of the score in the high-intermediate risk cohort was lower than the low-risk cohort, which can be explained by the greater heterogeneity in the risk profile of these individuals. It should also be noted that 23% of the patients belonging to this cohort had a risk of > 20%, an unusual proportion in similar studies^29,30^.

Although the discrimination of the ACC/AHA ASCVD score in the intermediate-high risk cohort was lower, it is similar to that reported in the literature^31^. In derivation cohorts, the area under the ROC curve was 0.7–0.74 in men by race and 0.81 in women regardless of race^32^. A study of multiethnic cohorts reported a C statistic of 0.71 for both sexes^31^, and validation studies in independent cohorts showed lower performance with a C statistic that fluctuated between 0.56 and 0.77^33–35^. This indicates that our results are comparable and even show better discrimination than other published studies.

Based on the results of the study, the use of a correction factor of 0.95 to the risk estimate (expected/observed event ratio of 1.05) is suggested for a more precise application of the score in patient populations similar to those included.

Muñoz et al. found an expected/observed relationship of 1.31 with a conversion factor of 0.75 when evaluating the calibration of the Framingham score in Colombia^17^. However, this conversion only applies to their low-risk groups since in the high-risk patients the score was not calibrated (expected/observed ratio 17.4)^17^.

Recently, the World Health Organization (WHO) released a calibrated and validated risk prediction model to estimate cardiovascular disease risk in 21 global regions. A chart to estimate risk in central Latin America was included. However, there is no external validation in Colombian population^36^.

Regarding the diagnostic performance of the ACC/AHA ASCVD score, for a cut-off point of 20%, it was found that the score correctly classifies individuals who are not at high risk. A patient classified as low risk has a low probability of developing a cardiovascular event within the next ten years.

This study showed that the ACC/AHA ASCVD risk score is calibrated and adequately discriminates the risk of cardiovascular events within ten years. The score maintains its operational performance both in a cohort with a low-risk profile and in a cohort with an intermediate/high-risk profile. This suggests that the ACC/AHA ASCVD risk score is a better tool for the estimation of cardiovascular risk in the Colombian population than the Framingham score. These findings will allow the application of guidelines recommendations that imply the use of this score for clinical decision-making.

### Strengths and weaknesses

This study is the first to validate the ACC/AHA ASCVD cardiovascular risk scale in a Latin American population cohort. It should be noted that no patient was taking a statin at the time of inclusion; thus, reducing the bias in the estimation of cardiovascular risk typical of this treatment. However, a general weakness of long-term prediction models is the modification of the risk profile after risk has been assessed, due to the new onset of risk factors or interventions. Another strength of the study was that 100% of the included patients had a 10-year follow-up, allowing greater certainty of the presented events without the need for imputations. Patients excluded because of loss of follow-up did not have a significant difference in calculated cardiovascular risk compared to patients with a complete follow-up, so they would not be expected to have a higher proportion of events.

A differential aspect compared to other works was the evaluation of the model both in a low-risk cohort and in an intermediate/high-risk cohort. This enabled us to describe and compare the diagnostic performance of the score for each cohort separately, revealing significant robustness in our result. The ACC/AHA ASCVD score shows better performance than the previously validated Framingham score, a finding which will modify the usual clinical practice in the Colombian population regarding the evaluation of cardiovascular risk in the primary prevention setting. However, the inclusion of a low-risk sample led to having less than 10 outcomes per variable in this specific cohort, which was expected due to the low calculated cardiovascular risk of this population. Although this may lead to imprecise results, the analyses are consistent and coherent across the cardiovascular risk spectrum.

The most important limitation of the study is the minimal representation of the black race in the included population. Less than 1% of included patients with available information on race self-identified as Afro-Colombian. This is due to the characteristics of the population served at our institution. However, several sensitivity analyses were carried out with the race variable, without finding changes in the results. The authors acknowledge that the poor representation of the Afro-Colombian race may mean a bias when estimating the risk in this racial group, so they recommend using the score with caution in this and other racial groups in Colombia and Latin America.

Being a study conducted in a single center, is another limitation to consider. The population included in the study could not be a representative sample of the Colombian population, because of the cultural heterogeneity and differences in health access and risk factors between regions. However, our study includes individuals with different socioeconomic status and risk factor patterns since it derives from two different cohorts. Another limitation of the study was the type of data collection, we conducted a retrospective cohort study selecting exclusively outpatients rather than a wider community study, thus limiting its generalizability. However, this is overwhelmed with the inclusion of two different cohorts of patients followed by 10 years in 100% of cases.

Although the number of cardiovascular events reported in both cohorts was low, it matches with the numbers of expected events. The authors do not recognize this issue as a limitation of the study, rather a strength of the scale.

## Conclusions

As recommended by the American Heart Association, an external validation study of the ACC/AHA ASCVD cardiovascular risk score was carried out to assess its diagnostic performance in a population not included in the original model. It is the first study carried out on a Latin American population. The score is calibrated, and its discrimination is good across the cardiovascular risk spectrum in a primary care setting. This model has operational characteristics that favor the use of the estimation of cardiovascular risk for the next ten years in the population studied. This is the first step to better classify cardiovascular risk in Latin American cohorts. This tool can be used in clinical practice for therapeutic decision-making. However, further validation studies are required with larger patient samples involving greater racial diversity.

## Supplementary Information

Supplementary Information.

## Data Availability

The datasets analyzed during the current study are available from the corresponding author on reasonable request.

## Abbreviations

CVD: Cardiovascular disease
ASCVD: Atherosclerotic cardiovascular disease
LDL: Low density lipoprotein
FSFB: University Hospital Fundación Santa Fe de Bogotá
HDL: High density lipoprotein
HL: Hosmer–Lemeshow
IQR: Interquartile range
ROC: Receiver operating characteristic
AMI: Acute myocardial infarction
