# Supplementary material for: External validation of the ACC/AHA ASCVD risk score in a Colombian population cohort
Source: Sci Rep. 2023 Apr 15;13:6139. doi: 10.1038/s41598-023-32668-4 (PMC10105759; doi:10.1038/s41598-023-32668-4)
Supplement: Supplementary file 1 — Supplementary Information. [file 41598_2023_32668_MOESM1_ESM.docx]

**Additional file 1**

| TABLE S1 Baseline characteristics of the patients included in the study and the patients lost to follow-up at 10 years. | | | |
| --- | --- | --- | --- |
| **Characteristic** | **Included patients (n=918)** | **Lost to follow-up at 10 years (n=465)** | **p-value** |
| Age (years), median (SD) | 51,7 (9,2) | 50,5 (8,2) | 0,01** |
| Male sex, n (%) | 589 (64,2) | 322 (69,2%) | 0,06* |
| Afrocolombian race, n (%) | 9 (1) | 1 (0,2) | 0,11* |
| Smoking, n (%) | 91 (9,9) | 50 (10,7) | 0,63* |
| Diabetes mellitus, n (%) | 113 (12,3) | 20 (4,3) | <0.001* |
| Antihypertensive treatment, n (%) | 235 (25,6) | 67 (14,4) | <0.001* |
| TC (mg/dl), median (SD) | 206,7 (34) | 214,4 (43,4) | <0.001** |
| HDLc (mg/dl), median (SD) | 45,8 (12,1) | 44,5 (11) | 0,06** |
| SBP (mmHg), median (SD) | 122,4 (17,1) | 124,6 (17,8) | 0,02** |
| Overweight, n (%) | 409 (44,5) | 180 (38,7) | 0,09* |
| Obesity, n (%) | 150 (16,3) | 96 (20,6) | 0,09* |
| CKD, n (%) | 14 (1,5) | 1 (0,2) | 0,03* |
| CID, n (%) | 26 (2,8) | 3 (0,6) | 0,007* |
| AHA/ASCVD score, median (IQR) | 3,6 (1,7-8,5) | 3,6 (1,7-7,4) | 0.99*** |
| TC = total cholesterol, HDLc = high density lipoprotein cholesterol, SBP = systolic blood pressure, CKD = chronic kidney disease, CID = chronic inflammatory diseases, CV = cardiovascular  *p-value by X2. ** p-value by t-Student. *** p-value by Mann Whitney. | | | |
